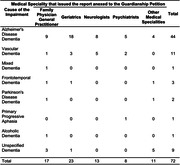# Analysis of medical reports of guardianship proceedings of people with dementia a sample from Belo Horizonte, Minas Gerais, Brazil

**DOI:** 10.1002/alz.092606

**Published:** 2025-01-09

**Authors:** Mariana S L C Real, Izabela G Barbosa, Carla V Carvalho, Marco Aurelio Romano‐Silva, Maria Aparecida Camargos Bicalho, Edgar Nunes de Moraes, Bernardo M Viana

**Affiliations:** ^1^ Older Adult’s Psychiatry and Psychology Extension Program Federal University of Minas Gerais, Belo Horizonte, Minas Gerais Brazil; ^2^ Geriatrics and Gerontology Center Clinical Hospital of University of Minas Gerais, Belo Horizonte, Minas Gerais Brazil; ^3^ Department of Psychiatry, School of Medicine, Federal University of Minas Gerais, Belo Horizonte, Minas Gerais Brazil; ^4^ School of Law, Federal University of Minas Gerais, Belo Horizonte, Minas Gerais Brazil; ^5^ Older Adult’s Psychiatry and Psychology Extension Program (PROEPSI), School of Medicine, Universidade Federal de Minas Gerais (UFMG), Belo Horizonte, Minas Gerais Brazil; ^6^ National Institute of Science and Technology Neurotec R (INCT‐MM), Belo Horizonte, Minas Gerais Brazil; ^7^ Cog‐Aging Research Group, Universidade Federal de Minas Gerais (UFMG), Belo Horizonte, Minas Gerais Brazil; ^8^ National Institute of Science and Technology (INCT‐Neurotec R), Faculty of Medicine, Federal University of Minas Gerais, Belo Horizonte, Minas Gerais Brazil; ^9^ Department of Internal Medicine, School of Medicine, Federal University of Minas gerais, Belo Horizonte, Minas Gerais Brazil

## Abstract

**Background:**

Dementia syndromes are chronic health conditions that lead to significant cognitive decline and functional impairment, including acts of civil life. Concerning the latter, a guardianship petition maybe needed when patients or family are at risk.

**Methods:**

Retrospective cross‐sectional study of documentary research on 72 electronical guardianship proceedings involving adults with dementia. The study sample comprises reports from six out of twelve Family Courts within the Court of Justice of Minas Gerais, with twelve reports collected from each selected court. The results were analyzed using descriptive statistics, involving mean, standard deviation, median, minimum and maximum. For statistical analyzes the software SPSS Version 20.0 for Microsoft Windows was used. When comparing continuous variables, the non‐parametric Mann‐Whitney test and the Kruskal‐Wallis test were used. The study was approved by the ethics committee by Federal University of Minas Gerais.

**Results:**

The sample had median age of 82.5 years with high level of education by Brazilian standards with a median of 8 years, and mainly composed by elderly women (68,1%) and widows (41,7%). Notably, 76.4% of guardianship proceedings were petitioned by sons, with predominance of daughters appointed as guardians. Geriatrics and Family Physicians (54.1%) stood out as the specialties that issued the majority of the initial medical reports. Alzheimer’s Disease Dementia (ADD) (61.1%) was the most prevalent diagnosis with a predominance of women (67.3%) and older people with a mean of 84.9 years (SD 8,6). In second place, vascular dementia (VD) (15.3%) followed by unspecified etiology of dementia (12.5%). In the non‐Alzheimer dementia group, the median age was 79.5 years (p = 0.016). In relation to education, there was no statistically significant difference between the group of patients with Alzheimer’s disease and non‐Alzheimer’s disease (p = 0.547).

**Conclusions:**

The present work identified that the majority of medical reports were related to ADD and VD and were made by geriatricians and family physicians. This points to the importance of these specialties regarding the protection and promotion of rights of people with these dementias.